# Indigenous knowledge of medicinal plants used by Saperas community of Khetawas, Jhajjar District, Haryana, India

**DOI:** 10.1186/1746-4269-6-4

**Published:** 2010-01-28

**Authors:** Manju Panghal, Vedpriya Arya, Sanjay Yadav, Sunil Kumar, Jaya Parkash Yadav

**Affiliations:** 1 Department of Genetics, M.D. University Rohtak, Haryana, India; 2 Department of Environment Science, M.D. University Rohtak, Haryana, India

## Abstract

**Background:**

Plants have traditionally been used as a source of medicine in India by indigenous people of different ethnic groups inhabiting various terrains for the control of various ailments afflicting human and their domestic animals. The indigenous community of snake charmers belongs to the 'Nath' community in India have played important role of healers in treating snake bite victims. Snake charmers also sell herbal remedies for common ailments. In the present paper an attempt has been made to document on ethno botanical survey and traditional medicines used by snake charmers of village Khetawas located in district Jhajjar of Haryana, India as the little work has been made in the past to document the knowledge from this community.

**Methods:**

Ethno botanical data and traditional uses of plants information was obtained by semi structured oral interviews from experienced rural folk, traditional herbal medicine practitioners of the 'Nath' community. A total of 42 selected inhabitants were interviewed, 41 were male and only one woman. The age of the healers was between 25 years and 75 years. The plant specimens were identified according to different references concerning the medicinal plants of Haryana and adjoining areas and further confirmation from Forest Research Institute, Dehradun.

**Results:**

The present study revealed that the people of the snake charmer community used 57 medicinal plants species that belonged to 51 genera and 35 families for the treatment of various diseases. The study has brought to light that the main diseases treated by this community was snakebite in which 19 different types of medicinal plants belongs to 13 families were used. Significantly higher number of medicinal plants was claimed by men as compared to women. The highest numbers of medicinal plants for traditional uses utilized by this community were belonging to family *Fabaceae*.

**Conclusion:**

This community carries a vast knowledge of medicinal plants but as snake charming is banned in India as part of efforts to protect India's steadily depleting wildlife, this knowledge is also rapidly disappearing in this community. Such type of ethno botanical studies will help in systematic documentation of ethno botanical knowledge and availing to the scientific world plant therapies used as antivenin by the Saperas community.

## Background

Utilization of plants for medicinal purposes in India has been documented long back in ancient literature because they are essential to human survival [1,2]. The consumption, management and valuation of wild plants are central aspects of the traditional knowledge in many human populations. Thus, plants gathering, the diffusion and conservation of knowledge within the community are traditional practices that have contribution to the subsistence of many cultures. In most of the societies the medical system coexists with several traditional systems. These traditional medical systems are generally based on the uses of natural and local products which are commonly related to the people's perspective on the world and life [3].

In India, there are about 54 million indigenous people of different ethnic groups inhabiting various terrains. These indigenous groups possess their own distinct culture, religious rites, food habit and have a rich knowledge of traditional medicine [4-8]. Even today, indigenous and certain local communities practised herbal medicine to cure a variety of diseases, with plants particularly used as folk medicine to treat snakebites [9-11]. Traditional herbal medicine is readily available in rural areas for the treatment of snakebite. Application of the plant or its sap onto the bite area, chewing leaves and bark or drinking plant extracts or decoctions are some procedures intended to counteract snake venom activity. Plants are used either single or in combination, as antidotes for snake envenomation by rural populations in India and in many parts of the world [12].

Snake charmers belong to the 'Nath' community living in this study area frequently use drugs prepare from medicinal plants found in the area for the treatment of snake bite victims. The community has also extensive knowledge about medicinal herbs which they gathered during their trips to the forest to trap snakes, and while roaming from one place to another place they dispense their herbal knowledge to their costumers. For centuries, snake charmers were enduring symbols of India. But the community has been virtually forgotten in a modernizing country and also due to ban of snake charming profession as part of efforts to protect India's steadily depleting wildlife. Hence, the aim of the present study was to document and analyze medicinal plants knowledge of the Saperas ethnic group, which they use for the treatment of snakebite and other type of diseases.

## Description of Study site

**Figure 1:**
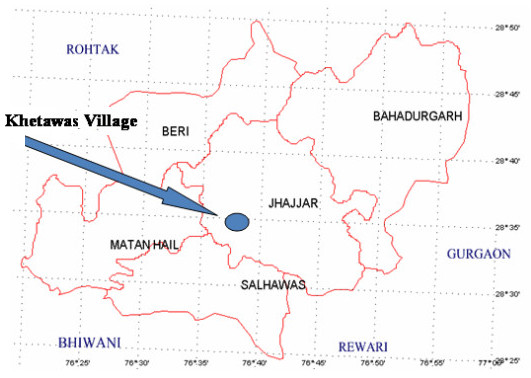
**Map of the district Jhajjar showing the study area**.

The study was carried out from a prominent village of Saperas community Khetawas located about 20 Km. from district headquarter, Jhajjar in Haryana, India (Figure 1). The district lies between 28° 33' N and 28° 42' S latitude and 76° 28' 45" W and 76° 84' 15" E longitude. The district is having an area of 1834 square Kms which is 4.05% of total area of the Haryana state. The total population of the District was 880072, (684975 in Rural and 195097 in Urban Areas) as per the Census record of 2001[13]. Rural population comprised 77.83% of the total population. Population density is 484 people per sq. km. The altitude of the district is about 715 above mean sea level (MSL) and a slope from South to North from Rewari towards Jhajjar is around 40 feet. In the eastern part of district, the area is considerably even. Some area is uneven and also suffers from inundation and water logging during Monsoon season. The district falls within the classified arid and semi-arid zones. Broadly four types of soil are available in the District viz. clay, loamy clay, loamy and sandy. However, the soil is deficient in Nitrogen. Hot summer, cold winter and meager rain fall are the main climatic characteristics of Jhajjar District. Two hospitals, 18 public health centers and 8 dispensaries are present in districts. Khetawas village having a total population of about 3000 peoples, out of which about 200 families of the Saperas community lived in the village and almost every family engaged in work of traditional healer. Out of these traditional healers 8 to 9 persons are the most popular for treatment of snakebite in the Haryana state. Yearly about 50 to 100 peoples come to these healers for treatment of snakebite. The numbers of persons are higher in rainy season because people encountered snake mainly in rainy season.

## Materials and methods

### Methods of informants and data collection

In order to document the utilization of indigenous medicinal plants, survey was carried out during the year, July 2008 to August 2009. The information on medicinal uses of the indigenous plants have been described after gathering information's from experienced rural folk, traditional herbal medicine practitioners who were having knowledge of traditional healing. A total of 42 selected inhabitants were interviewed. Out of 42, 41 were male and only one woman. The age of the healers was between 25 years and 75 years. A brief group discussion was made with the informants in local language, i.e. Haryanvi (a dialect of Hindi) prior to ethnobotanical data collection to get there consent and to explain to them that their cooperation is a valuable contribution to the documentation of the traditional plant used by them. In addition direct plant field observations were employed to collect the data on the knowledge and management of medicinal plants with the help of local healers known as 'Naths'. Maximum numbers of medicinal plants used by the healers were collected from Jhajjar District and in its nearby villages. A structured questionnaire was used to elicit information from the resource persons using standard methods [14]. The data collection Performa has been given as Additional file 1. Information on local name of the plant, plant parts used for curing disease, their recipes and mode of administration were recorded. From the collected data a list of plants of different families with their traditional uses, plant part used, their recipes and mode of administration is prepared in alphabetical order of disease treated and along with the name of the plants. We did not use any "statistical survey" in this study.

### Identifications of plants

The collected plants were identified in the laboratory and further confirmation was made by Prof. S. Biswas, Head, Department of Botany, Forest Research Institute, Dehradun, India and the specimens of the plants were compared with DD herbarium, Dehradun. Comparison of flora was also made according to different references concerning with the medicinal plants of Haryana and adjoining areas [15-18]. The voucher specimens were deposited in the herbarium of Genetics Department, M.D. University, Rohtak.

### Plant categorization and abundance of the plant species

Plants were classified in the categories of wild or cultivated and also classified into different types of growth forms (herbs, shrubs and trees). Abundance is the number of individuals of any species per sampling unit. The abundance of medicinal plants in the study area was calculated on the basis on methods mentioned by Chaudari and Sarkar [19]. The abundance was categorized as under:

S = Sporadic i.e. growing scattered; need careful monitoring.

T = Threatened i.e. the species are taken care of for conservation.

PS = Presently safe but need effort to protect them.

D = Doubtful presence

## Result and discussion

### Medicinal plants reported

The information's on scientific name, common name, family name, habit, ailment treated, voucher specimen number, status and abundance of plants have been shown in Table 1. Type of disease treated, application route, mode and methods of application of drugs has been shown in Additional file 2. The study revealed that the healers of the snake charmer community used 57 medicinal plants species that belonged to 51 genera and 35 families. The study has brought to light that the major emphasis of this community was employed in the treatment of snakebite. It was reported that 19 plants belongs to 13 families were widely used as snakebite remedies and 48 plants belongs to 34 families were used in the treatment of other diseases. According to habit of plants, 20 were herbs (36%), 16 trees (28%), 10 climbers (18%), 9 shrubs (16%) and one creeper (2%). The common use of herbaceous medicinal plants was also reported in other parts of world [20-22]. In the present study the most represented family with highest number of utilized medicinal plants in the area was Fabaceae (8 plants) followed by Liliaceae (5 plants), Laminaceae and Asteraceae (3 plants each). Thirty six (37.03%) plants were categorized as wild plants and 20(37.03%) as cultivated plants. The study of abundance of plant data reveled that 27(48.02%) were presently safe, 19 (33.92%) sporadic, 7(12.5%) threatened and status of 3(5.35%) plants was not known.

**Table 1:** Characteristics of medicinal plants used by the snake charmers

S. No.	Botanical name	Vernacular name	Family name	Habit	Ailment treated	Voucher number	Status	Abundance
1	*Acacia arabica *(Lam.) willd.	Kikar	Fabaceae	T	Cough, jaundice, male fertility disorders	MDU 2601	W	PS
2	*Achyranthes aspera *L.	Ola kanta	Amaranthaceae	H	Snake bite, tooth ache	MDU 6001	W	PS
3	*Acacia catechu *(L.f.) Willd	Kher	Fabaceae	T	Mouth ulcers	MDU 2608	W	S
4	*Aegle marmelos* (L.) Correa Ex. Schultz	Bael Patthar	Rutaceae	T	Abdomen disorders, diabetes	MDU 155630	W	PS
5	*Allium cepa *L.	Piyaz	Liliaceae	H	Fever, snake bite	MDU 6801	C	PS
6	*Albizia lebbeck *(L.) Benth.	Sirus	Fabaceae	T	Eye diseases, male fertility disorders, snake bite	MDU 2604	W	PS
7	*Allium sativum *L.	Lasan	Liliaceae	H	Microbial contaminations,	MDU 6802	C	PS
8	*Aloe vera *(L.) Burm.f.	Guarka-patha	Liliaceae	H	Abdomen disorders, piles	MDU 6803	C	S
9	*Argemone mexicana *L.	Kateli	Papaveraceae	H	Female sex disorders, eye diseases, mental disorders, skin diseases, tooth ache, wound healing	MDU 401	W	PS
10	*Artemisia scoparia *Waldst. & Kit.	Nagdman	Asteraceae	H	Snake bite	MDU 3802	W	S
11	*Asparagus racemosus *Willd.	Arra Kanta	Liliaceae	CL	Fever	MDU 6806	C	T
12	*Azadirachta indica *A. Juss	Neem	Meliaceae	T	Allergy, skin diseases, snake bite	MDU 1801	C	PS
13	*Brassica campestris *L.	Kali sarson	Brassicaceae	H	Abdomen disorders, allergy	MDU 507	C	PS
14	*Barleria cristata *L.	Kala bansa	Acanthaceae	S	Cough	MDU 5606	W	S
15	*Butea monosperma *(Lam.) Taub.	Dhak	Fabaceae	T	Snake bite	MDU 2404	W	S
16	*Bryophyllum calycinum *Salisb.	Pattarchat	Crassulaceae	S	Wound healing	MDU 12001	W	PS
17	*Capparis aphylla *Roth.	Kair	Capparidaceae	T	Piles	MDU 601	W	T
18	*Cassia fistula *L.	Amaltas	Fabaceae	T	Skin diseases, snake bite	MDU 2503	W	PS
19	*Cassia obtusifolia *L.	Sonmakhi	Fabaceae	S	Eye diseases	MDU 2518	W	D
20	*Calotropis procera *(Ait) R. Br.	Aak	Asclepiadaceae	S	Abdomen disorders, allergy, cough, fever, fistula, eye diseases, male fertility disorders, skin diseases, snake bite, wound healing	MDU 4602	W	S
21	Cannabis sativa L.	BhangBhang	Cannabinaceae	H	Mental disorders, snake bite	MDU 6501	C	S
22	*Cassia occidentalis *L.	Kasaundi	Fabaceae	S	Snake bite	MDU 2504	W	S
23	*Citrullus colocynthis *(L.) Schrad.	Gadumba	Cucurbitaceae	CL	Snake bite	MDU 3301	W	S
24	*Cordia dichotoma *Forst. L.	Lesua	Boraginaceae	T	Mouth ulcers	MDU 4801	W	S
25	*Cocculus villosus *DC.	Nagdun	Menispermaceae	CL	Fistula, snake bite	MDU 301	W	S
26	*Curculigo capitulata *Gaertn.	Kali musli	Liliaceae	H	Female sex disorders	MDU 6703	C	T
27	*Curcuma longa *L.	Haldi	Zingiberaceae	H	Female sex disorders, eye diseases, wound healing	MDU 8001	C	T
28	*Cuscuta reflexa *Roxb.	Amerbel	Convolvulaceae	CL	Fever,	MDU 5001	W	PS
29	*Cyperus rotundus *L.	Motha	Cyperaceae	H	Microbial contaminations	MDU 7001	W	PS
30	*Datura metel *L.	Dhatura	Solanaceae	S	Cough, male fertility disorders, mental disorders, respiratory problems	MDU 5103	W	S
31	*Eclipta alba *(L.) Hassk.	Bhringraj	Asteraceae	H	Snake bite	MDU 3803	W	S
32	*Emblica officinalis *Gaertn.	Amla	Euphorbiaceae	T	Eye diseases, jaundice	MDU 6203	C	PS
33	*Eugenia jambolana *Lam.	Jamun	Myrtaceae	T	Diabetes	MDU 2902	W	PS
34	*Ficus benghalensis *L.	Badd	Moraceae	T	Cough, diabetes fistula, jaundice, male fertility disorders, snake bite, tooth ache, wound healing	MDU 6401	W	PS
35	*Gloriosa superba *L.	Kalihari	Colchicaceae	CL	Snake bite	MDU 6813	C	T
36	*Kyllinga monocephala *Rottb	Safad Nirbashi	Cyperaceae	CR	Snake bite	MDU 7010	W	D
37	Leucas cephalotes Spreng	Goma	Lamiaceae	H	Snake bite	MDU 5802	W	PS
38	*Mangifera indica *L.	Aam	Anacardiaceae	T	Skin diseases	MDU 2301	W	PS
39	*Melia azadirachta *L	Bakain	Meliaceae	T	Microbial contaminations, piles	MDU 1802	C	S
40	*Mesua ferrea *L.	Nag kesar	Clusiaceae	T	Mental disorders	MDU 11001	C	T
41	*Mimosa pudica *L.	Chui-mui	Fabaceae	H	Diabetes	MDU 2605	W	PS
42	*Momordica balsamina *L.	Jangli kerala	Cucurbitaceae	CL	Snake bite	MDU 3314	W	PS
43	*Momordica dioica *Roxb. (Ex willd.)	Banj kerala	Cucurbitaceae	CL	Female sex disorders, male fertility disorders	MDU 3304	W	S
44	*Ocimum basilicum *L.	Marua	Lamiaceae	H	Fever,	MDU 5807	C	PS
45	*Ocimum sanctum *L.	Tulsi	Lamiaceae	H	Male fertility disorders, skin diseases, snake bite	MDU 5804	C	PS
46	*Opuntia dillenii *(Ker-Gawl.) Haw.	Nagphani	Cactaceae	S	Fistula	MDU 3401	W	PS
47	*Oroxylum indicum *(L.) Vent.	Aralu	Bignoniaceae	T	Respiratory problems	MDU 5306	W	S
48	*Pedalium murex *L.	Vilayati gokhru	Pedaliaceae	H	Male fertility disorders	MDU 5401	W	PS
49	*Peperomia pellucida *(L.) Kunth.	Panpatta	Piperaceae	H	Fistula	MDU 901	C	D
50	*Punica granatum *L.	Anar	Punicaceae	S	Female sex disorders, jaundice	MDU 3101	C	S
51	*Raphanus sativus *L.	Muli	Brassicaceae	H	Piles	MDU 503	C	PS
52	*Solanum ferox *L.	Lakshamana	Solanaceae	H	Female sex disorders	MDU 5115	W	T
53	*Spilanthes acmella *Murr.	Akarkara	Asteraceae	H	Cough	MDU 3808	C	S
54	*Tinospora cordifolia *(Willd.) Miers. ex. Hook. F. & Thoms	Giloy	Menispermaceae	CL	Fever, jaundice	MDU 302	C	T
55	*Tribulus terrestris *L.	Deshi gokhru	Zygophyllaceae	CL	Female sex disorders	MDU 1301	W	PS
56	*Tylophora indica *(Burma.L.) Merr.	Anta mul	Asclepiadaceae	CL	Female sex disorders	MDU 4612	W	PS
57	*Withania somnifera *(L.) Dunal	Aksin	Solanaceae	S	Cough	MDU 5111	W	S

Abundance status (C = Cultivated, D = Not Known, PS = Presently Safe; S = Sporadic = T = Threatened, V = vulnerable species, Endangered species = EN)

Habit (CL = Climber, CR = Creeper, H = Herb, S = Shrub, T = Tree)

Status of plants (W = wild, C = Cultivated)

### Plant parts used and mode of remedy preparations

In most of the preparations leaves (27%) were used for the preparation of medicines predominantly followed by roots (23%), fruits (10%), seeds (10%), stem barks (9%), whole plant (7%), latex (6%), root bark (4%), flower (3%) and gum (1%). The common use of leaf in the preparation of remedies could partly be due to the relative ease of finding this plant part. Leaves remain green and available in plenty for the most months of the years. The use of leaves in the preparation of remedies is also common elsewhere [21,23-27]. The common use of leaf is also due to easily availability of this plant parts in the area. The most prevalent methods of drug preparation were as infusion (23%), powder (16%), decoction (10%) and paste (10%). Remedies were seldom prepared as pellets (9%), juice (6%), band (2%) and fumes (2%). The use of water as dilutant was the most frequently found for the preparation of drug, other useful dilutant were reported oil, butter and cow milk. Oils from *Ricinus communis*, *Seasamum indicum*, *Brassica juncea *and *Azadirachta indica *were mixed with plant medicine as dilutant. The mixing of oil of these four plants for preparation of drugs was also reported in Kani tribals of Tamil Nadu [27]. Healers of Saperas community also mix sugar in herbal formulation and similar results were reported in a study from Kurukshetra Districts, Haryana [28]. The healers of Saperas community also use latex of *Ficus benghalensis *for mixing of various ingredients. It was reported that the healers prescribed the medicine either based on single plant parts or a combination of several plant parts and similar results were also reported in various studies conducted in Haryana [28] and other parts of India[23,27].

During the survey it was found that the healers of this community collect medicinal plants from variety of habitats. Mainly wild plants were collected from nearby Matanhail Bani (Reserve forest area) which is dominated by *Salvadora *trees. As Haryana is an agricultural state with low diversity of forest area so these peoples also collect medicinal plants from agricultural land, barren land and banks of canals. For the preparation of drugs the healers mainly use two methods. In the first method, drug preparation was done by shade drying and then pounding of the plant to form powder. The infusion or decoction of this powder is prepared after boiling with water. In the second method, pellets were prepared after mixing with Cow's Ghee (clarified butter) or with other lubricant like oil of plants.

### Route of administration and dosage

Sixty three percent of the healer remedies were applied through oral tract while 23% were applied on the skin and 6% administrated through the eyes. Few remedy preparation were applied topically in mouth (5%) and some through the nasal tract (3%). For the treatment of snake bite, 80% remedies were applied through oral route in form of infusion or decoction and 20% were applied topically on the snake bite area. Most treatments were reported to be completed within two or three days. Majority of drugs recommended for thrice a day. But, in case of snake bite treatment these healers kept the patients for two or three days under continuous observation till the patients were antivenin. The patients were considered antivenin if the drug remedies (mixture of leaves and roots of some plants) taste bitter but if the drug is sweet to taste, the patients needs urgent attention of healers. Dosage was repeated until the taste return to normal (Additional file 2). Liquid remedies administrated to patients were usually measured by spoon or cup or number of drops. When patients did not show any sign of recovery to their diseases than the healers send the patients to nearby modern health centers.

### Medicinal plant Knowledge secrecy, mode of transfer and Threats

Elder people (80% above age of 50 years) mentioned and utilized more variety of medicinal plants compared to younger generation. The name and age of the informants have been given in Table 2. Women of this community have very little knowledge of medicinal plants. Similarly, literate person of the area were found to have less knowledge of medicinal plants as compared to illiterate ones due to lack of their interest. It was also noted that 80% people of this community were hesitant in disclosing their knowledge. They fear that their recognition in the society which they have earned due to their knowledge will be lost and hence they want to keep it secret. The traditional knowledge acquired from their ancestors is freely transferred within the family preferably to the eldest son that's why the male generation of this community has a rich traditional knowledge of medicinal plants. They were ready to transfer of this knowledge to the outside world only on the basis of substantial payment. The secrecy of traditional medical practice is also a common phenomenon found in other part of Haryana [29], India [23] and worldwide [24,27]. Reputed healers of this community do not keep records and the information is mainly passed on verbally from generation to generation. This knowledge is however dwindling rapidly due to changes towards a more western lifestyle, modern agricultural practices, cultural changes within the community, rapid shift towards the allopathic medicine, housing colonies and modern education lead to the destruction of not only the habitats of medicinal plants but also vanishing of traditional knowledge and medicinal plant species are threatened day by day in the area. Similarly the threat to traditional knowledge also observed in other parts of India due to less interest of the younger generation [21].

**Table 2:** Name and age of the informants

S. No.	Botanical names	Name of Informants(Age)
1	*Acacia arabica *(Lam.) willd.	Rajunath(45 years)
2	*Achyranthes aspera *L.	Omnath(39) years), Rajunath(45 years)
3	*Acacia catechu *(L.f.) Willd.	Rajunath(56 years), Bijendernath(68 years)
4	*Aegle marmelos *(L.) Correa Ex. Schultz	Rajunath(55 yaers)
5	*Allium cepa *L.	Omnath(42 years)
6	*Albizia lebbeck *(L.) Benth.	Somnath(74 years)
7	*Allium sativum *L.	Anujnath(32 years)
8	*Aloe vera *(L.) Burm.f.	Rameshnath(59 years)
9	*Argemone mexicana *L.	Omnath(42 years)
10	*Artemisia scoparia *Waldst. & Kit.	Rajunath(45 years), Tulsinath(68 years)
11	*Asparagus racemosus *Willd.	Sureshnath(59 years)
12	*Azadirachta indica *A. Juss	Omnath(42 years)
13	*Brassica campestris *L.	Arjunnath(53 years)
14	*Barleria cristata *L.	Omnath(62 years)
15	*Butea monosperma *(Lam.) Taub.	Bijendernath(68 years)
16	*Bryophyllum calycinum *Salisb.	Shambunath(72 years)
17	*Capparis aphylla *Roth.	Sajanath(66 years)
18	*Cassia fistula *L.	Sandeepnath(29 years)
19	*Cassia obtusifolia *L.	Omnath(26 years)
20	*Calotropis procera *(Ait) R. Br.	Omnath(62 years)
21	*Cannabis sativa *L.	Omnath(62 years)
22	*Cassia occidentalis *L.	Vednath(62 years)
23	*Citrullus colocynthis *(L.) Schrad.	Omnath(42 years)
24	*Cordia dichotoma *Forst. f.	Manunath(58 years)
25	*Cocculus villosus *DC.	Omnath(42 years)
26	*Curculigo capitulata *Gaertn.	Gorakhnath(75 years)
27	*Curcuma longa *L.	Rajeshnath(68 years)
28	*Cuscuta reflexa *Roxb.	Vishnunath(56 years)
29	*Cyperus rotundus *L.	Sadhunath(74 years)
30	*Datura metel *L.	Radhaa (69 years)
31	*Eclipta alba *(L.) Hassk.	Omnath(42 years)
32	*Emblica officinalis *Gaertn.	Sajjan(35 years)
33	*Eugenia Jambolana *Lam.	Ranjannath(64 years)
34	*Ficus benghalensis *L.	Rajbir (60 years)
35	*Gloriosa superba *L.	Omnath (26 years)
36	*Kyllinga monocephala *Rottb	Omnath(42 years)
37	*Leucas aspera *Spreng	Sunder(52 years)
38	*Mangifera indica *L.	Rambhaj(60 years)
39	*Melia azadirachta *L.	Shumbu (67 years)
40	*Mesua ferrea *L.	Ramdiyanath(68 years)
41	*Mimosa pudica *L.	Jagannath(62 years)
42	*Momordica balsamina *L.	Ojasvnath(59 years), Somnath(74 years)
43	*Momordica dioica *Roxb. (Ex willd.)	Somnath(63 years), Somvati(52 years)
44	*Ocimum basilicum *L.	Raman(69 years)
45	*Ocimum sanctum *L.	Shaamnath(53 years)
46	*Opuntia dillenii *(Ker-Gawl.) Haw.	Kailashnath(73 years)
47	*Oroxylum indicum *(L.) Vent.	Suraj(43 years)
48	*Pedalium murex *L.	Omnath(68 years), Somnath(63 years)
49	*Peperomia pellucida *(L.) Kunth.	Vikram(62 yaers)
50	*Punica granatum *L.	Sukernath(57 years)
51	*Raphanus sativus *L.	Shaamnath(53 years)
52	*Spilanthes acmella *Murr.	Kedarnath(61 years)
53	*Solanum ferox *L.	Omnath(68 years)
54	*Tinospora cordifolia *(Willd.) Miers. ex. Hook. F. & Thoms	Somvati(52 years)
55	*Tribulus terrestris *L.	Raman(69 years)
56	*Tylophora fasciculata *Buch. Ham. ex Wight	Kedarnath(73 years)
57	*Withania somnifera *(L.) Dunal	Omverth(63 years)

### Medicinal plants for snakebite and other ailing diseases

**Figure 2:**
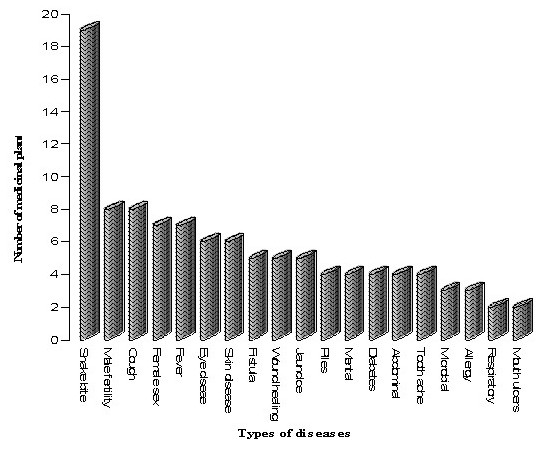
**Number of medicinal plants used for various diseases**.

**Figure 3:**
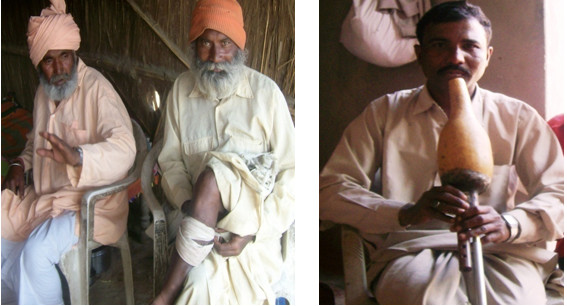
**Pictures of snake charmers**.

This community treated about 19 diseases ranging from abdominal disorders to wound healing (Additional file 2). Maximum numbers of plants were used for the cure of snake bite (19 plants), male fertility problems, cough (each treated with 8 plants) followed by female sex problems, fever (each treated with 7 plants), eye problems, and skin diseases (each treated with 6 plant species). Similarly for treatment of fistula, wound healing, jaundice (5 plants for each disease) and for piles, mental diseases, abdominal problems and tooth ache (4 plants for each) were used. Least number of plants (2 for each disease) was used by the healers for treatment of respiratory problems and mouth ulcers (Figure 2). The pictures of reputed Saperas community healers have been shown in Figure 3. The fact that higher proportion of medicinal plants besides the snake bite treatments were used by this community for male and female sex problems that could be attributed to the high prevalence of the disease in this area. We have reported that some plants were used in treatment of more than one disease. For example, different parts (leaves, stem bark, latex, and root bark) of *Calotropis procera *were found to be useful in the cure of 10 ailing diseases. *Argemone mexicana *has been found useful in the treatment of female fertility problems, eye diseases, mental disorders, skin diseases, tooth ache and wound healing. *Datura metel *has been found useful in cough, male fertility problems, mental disorders and respiratory problems. *Albizia lebbeck*, *Azadirachta indica *and *Curcuma longa *each of these were used for treatment of three ailing diseases. Herb like *Momordica dioica *was found to be useful in treatment of sex sterility both in male and female.

We have reported in our study that similar medicinal plant of different species was used by the healers of this community as used by the healers in different parts of India. For example the fruit juice of *Emblica officinalis *was used by the healers of Saperas community but other species *Phyllanthus amarus *was used by villagers of Dharapuram Taluk, Tamil Nadu for treatment of jaundice [30]. *Eclipta alba *was used by Saperas for the treatment of snake bite whereas the aqueous ethanolic extract of the aerial part of *Eclipta prostrata*, known as an antidote to snakebite in southern part of Tamil Nadu [12] and also used in other part of world like in Brazil and China, has been also tested against South American rattlesnake (*Crotalus durissus terrificus*) venom [31].

*Ficus benghalensis, Pedalium murex *and *Tribulus terrestris *were used to cure sexual diseases by the tribal healers of Southern Rajasthan [32]. Use of *Pedalium murex *and *Ficus benghalensis *for male sex problems were also reported in healers of Meo Community of Gurgaon, Haryana [29]. Same medicinal plants(*Ficus benghalensis, Pedalium murex *and *Tribulus terrestris*) were also used by the healers of Saperas community to cure sex problems. In our study the whole plant of *Cuscuta reflexa *was used as antiseptic to wound similar use of this plant was reported in Meo community of Gurgaon, Haryana [29]. *Ocimum sanctum *has a long Indian history of bearing an antitussive property but its analgesic use has been reported in this study. The analgesic use of *Ocimum sanctum *was also reported first time by Jaintia tribes in Assam [33]. Some medicinal plants used by Saperas community were also reported useful in the treatment of similar diseases as reported in Kurukshetra Districts, Haryana study [28] like use of *Achyranthes aspera *for skin diseases, *Cannabis sativa *and *Barleria cristata *for the treatment of cough, *Curcuma longa *as analgesic in fever, *Cassia fistula *to cure tooth ache and use of *Calotropis procera *to cure stomach pain.

Large numbers of plants along with different parts have been found to be effective as antidotes against snake venoms in various studies done in India [34-38]. In one another report *Gymnema sylvester *R.Br. (Asclepiadaceae) root and the whole plant of *Andrographis paniculata *Nees (Acanthaceae) are used against snakebites in folk medicine [39]. The root extract of *Vitex negundo *and *E. officinalis *having significant neutralizing capacity against *Viper russellii *and *Naja kaouthia *venom [38]. It is believed that triterpenoids present in *V. negundo *and *E. officinalis *involve in venom inactivation process. In a ethnobotanical study done in southern part of Tamil Nadu [12] some medicinal plants like *Eclipta prostrata*, *Achyranthes aspera *and *Gloriosa superba *were found to be useful in snakebite treatment and same plants were also reported in the present study by Saperas community. Several substances have been isolated from plants and tested against the lethal action of the venoms [31,40]. The fractions of wedaloactone (*Eclipta prostrate*), esters (*Gloriosa superb*) and glycocides (*Achyranthes aspera*) were found antivenom to snakebite [12].

So the data recorded during this study were compared with the related literature [[33,35,41,42], and [43]] and also recently published reports on the traditional medicinal uses of the plants [[27-29,31,44], and [45]]. It was found that some of these plants are already known for similar uses. However, their recipes, drug preparation methods, mode of use and addition of ingredients were different.

## Conclusion

It can be concluded from study that the snake charmers healers has highly specialized indigenous knowledge of medicinal plants. The medicinal plant resources of the region are diminishing due to over exploitation of certain species, illegally trading, laying of roads and other developmental works (that causes destruction of their habitats). As the people of this community inherit a rich traditional knowledge and documentation of this knowledge has provided novel information from the area. This will not only provide recognition of this undocumented knowledge but will also help in conservation of such rare, gradually vanishing important medicinal plants used for snake bite and other diseases. These highly interesting findings require further research, while the efficiency of the various indigenous practices will need to be subjected to pharmacological validation. Finally, we are advocating merely recording the use of plant products by a people in a little known region of India.

## Competing interests

The authors declare that they have no competing interests.

## Authors' contributions

All authors contributed equally during the field work, data analysis and preparation of the manuscript.

## Supplementary Material

Additional file 1 **Data collection Performa**. The data collection Performa represent the data acquisition questionnaire for utilization of medicinal plants, respondent consent agreement and researchers declaration. Click here for file

Additional file 2 **Description of diseases treated, parts used, application route, mode of preparation and administration of drugs used by the Saperas Community**. The data provided describe about the medicinal plants used by the healers for the treatment of other ailing diseases. Click here for file
