# Supplementary material for: Indigenous knowledge of medicinal plants used by Saperas community of Khetawas, Jhajjar District, Haryana, India
Source: J Ethnobiol Ethnomed. 2010 Jan 28;6:4. doi: 10.1186/1746-4269-6-4 (PMC2826346; doi:10.1186/1746-4269-6-4)
Supplement: Additional file 1 — Data collection Performa. The data collection Performa represent the data acquisition questionnaire for utilization of medicinal plants, respondent consent agreement and researchers declaration. [file 1746-4269-6-4-S1.PDF]

Data Collection Proforma

Data acquisition questionnaire for utilization of medicinal plants in Khetawas, Jhajjar District, Haryana, India

QUESTIONNAIRE

PART 1: INFORMANTS DETAILS

Name..Sex....M/F Age...........Years.

Occupation.. Level of education.......................................

Location/Residence...

Efficacy Data

Type of Disease treated ....

Type of Plant (Local name, Language used)...

Preparation method(s)...

Administration form (s)...

Part(s) of plant used ....

Route(s) of application...

Approximate dosage...

Response of Patient Good.........................Fair........................ Poor.........................

PART 2: RESPONDENTS CONSENT AGREEMENT

I...Hereby agree to participate in this study with my full consent and conscious and declare that to the best of my Knowledge the information that I have provided is true, accurate and complete.

Signature/Thumb print..Date............./ July /2008

PART 3: RESEARCHER'S DECLARATION

1. The following research will be undertaken with respect to the indigenous knowledge and intellectual proprietary of the Sapera Community.

2. We will at no given time initiate or conduct practices that are deemed to obtain information from the respondents by intimidation, coercion or false pretence.

3. The respondents will be informed of the intended project elaborately prior to questionnaire administration and in confidential to eliminate any degree of conspiracy.

4. We will be no under any obligation to edit or tamper the information provided by the respondents.

5. Translation and transcription will be necessary for clarification due to the language barrier.

6. The information collected will be used for the described research purpose and not any undisclosed any undisclosed intentions.

Signatory Researchers:

1. Manju Panghal

2. Ved Priya Arya

3. Sanjay Yadav

4. Sunil Kumar

5. Jaya Parkash Yadav
