# Supplementary material for: Indigenous knowledge of medicinal plants used by Saperas community of Khetawas, Jhajjar District, Haryana, India
Source: J Ethnobiol Ethnomed. 2010 Jan 28;6:4. doi: 10.1186/1746-4269-6-4 (PMC2826346; doi:10.1186/1746-4269-6-4)
Supplement: Additional file 2 — Description of diseases treated, parts used, application route, mode of preparation and administration of drugs used by the Saperas Community. The data provided describe about the medicinal plants used by the healers for the treatment of other ailing diseases. [file 1746-4269-6-4-S2.PDF]

Table 1. Description of diseases treated, parts used, application route, mode of preparation and administration of drugs used by the Saperas community

Disease cured	*Botanical name*	*Parts used*	*Application route*	*Mode of preparation and administration of the drug*
Abdomen disorders	*Aegle marmelos* (L.) Correa Ex. Schultz	F	Oral	The fruits (5 gm.) were pounded and mixed with isabgol (5 gm.), gum of *Acacia arabica* (5 gm.). 3 gm. of the mixture given with curd for two times a day to cure diarrhea.
	Aloe vera (L.) Burm.f.	R	Oral	The roots (10- 20 gm) were boiled for one hour and the decoction was given two times for two days to cure stomach pain.
	Brassica campestris L.	SE	Oral	Seeds were pounded and mixed with 1-2 mg of sugar. The resultant powder was recommended for licked to cure stomach pain.
	Calotropis procera (Ait) R. Br.	LE, SB	Oral	The leaves(10 gm.) and stem bark(1gm.) were pounded and mixed with cow milk (2ml.) Small sized pellets were made and 2 pellets were given per day for three days to cure sever stomach pain.
Allergy	*Azadirachta indica* A. Juss.	SE, LE	Oral	The seeds were pounded and also infusion of leaves prepared in water was given orally (2 ml) for two times in a day, for one month.
	Brassica campestris L.	SE	Topical	The warmed seeds oil (4ml.) was mixed with wax (3 gm.) and charcoal (4 ml.). Stir continually till it gets cool and apply the paste on skin for 1 week.
	Calotropis procera (Ait) R. Br.	La	Topical	The latex (6 drops) of plant mixed with *Ricinus cumminis* oil and applied topically to reduce irritation in the allergic skin. Overdose of latex can induce tenderness in skin.
Cough	*Acacia arabica* (Lam.) Willd.	G	Oral	The gum of *A. arabica* mixed with almond (5 gm.) and *Glycyrrhiza glabra* (2 gm.). These all were pounded together and pellets were made. These pellets were given two times a day for chewing.
	Barleria cristata L	FL, WP	Oral	The whole plant (20 gm.) was boiled with 50 ml. of water for 1 hour and filtered. Cow ghee (15 ml.) and flowers of *B. cristata s* (10 gm.) were mixed in the filtrate and boiled it again to make infusion. The 6 ml. of infusion was given orally for two times a day.
	Cannabis sativa L.	LE	Oral	Shade dried leaves were pounded and mixed with honey and oral administration was given with hot tea for 3 times in a day to cure cough.
	Calotropis procera (Ait) R. Br.	LE	Oral	The leaves (200 mg.) were pounded and mixed with jaggery (3 gm.) and orally administrated for two times a day for three days. Overdose of latex can cause vomiting.
	Datura metel L.	LE	Oral	The leaves (4gm.) were pounded and pellets were prepared by mixing with jaggery and leaves of *C. procera* (4gm.). One pellet in the morning and evening time was advised to take orally.
	Ficus benghalensis L.	SB	Oral	The stem bark pounded (10 gm.) with leaves of *A. Mexicana* (3 gm.) and powder was made. The powder was given with honey (5ml) for three times.
	Spilanthes acmella	LE	Oral	The leaves were pounded and boiled for 1 hour. The decoction (5 ml.) of

	Murr.			the leaves was given for six days to cure old cough
	Withania somnifera (L.) Dunal	R	Oral	The root (20 gm.) pounded and decoction was made by boiling in water for 2 hour. 25 gm. of sugar was added and given orally two times in a day. Over dose can cause uneasiness.
Diabetes	*Aegle marmelos* (L.) Correa Ex. Schultz	LE	Oral	The leaves were boiled and infusion (10 ml) was given in the morning to the diabetic patients.
	Ficus benghalensis L.	SB, LE	Oral	The stem bark and leaves were pounded and boiled for ½ hour with 1 lt. water of water. Filter the decoction and the filtrate (2ml) was given for 1 month in morning and evening.
	Eugenia Jambolana Lam.	SE	Oral	The seeds (4 gm.) were mixed with rhizome of *Zingiber officinale* (4 mg.) and whole plant of *Gymnema sylvestre* (10 gm.). All were pounded. From the resultant powder pellets were made with juice of *Aloe vera* and these pellets were administrated orally for three with honey times a day, before meal.
	Mimosa pudica L.	R	Oral	The roots were pounded and boiled with water and 10ml of infusion given orally for one month to the diabetic patients.
Female sex problems	*Curculigo capitulata* Gaertn.	R	Oral	The powdered rhizome was boiled with water and the decoction (10 ml.) given orally to the patient twice a day for a period of 10 -15 days for cure of vaginal infection.
	Curcuma longa L.	R	Oral	Turmeric extract(3 mg.) prepared after boiling with water given to female twice daily for two weeks prior to expected menstruation to cure menstrual problems
	Momordica dioica Roxb. (Ex willd.)	R	Oral	The rhizome of female plant pounded and dry powder (6 mg.) given to females for one month to cure female sterility.
	Punica granatum	R	Topical	The roots (5gm.) were pounded with root of *Argemone mexicana* (5gm.) and a paste was made and this paste was applied on the breasts to increase their tenderness.
	Solanum ferox L.	R	Oral	The fresh roots pounded with cow milk and the paste was made. 1mg. of this paste was given after fourth day of menstrual cycle to cure female infertility and for birth of a male child.
	Tribulus terrestris L.	F	Oral	The fruit (10-20 mg.) pounded and infusion was made after boiling with water. This infusion (1ml.) was taken orally for 1 week to cure female infertility.
	Tylophora indica (Burma. f.) Merr.	R	Oral	The powdered rhizome (2mg.) was mixed with sugar and milk and given to prevent abortion.
Fever	*Allium cepa* L.	R	Oral	A 20 mg piece of rhizome spreaded with black pepper and given to the patients twice a day.
	Asparagus racemosus Willd.	R	Oral	The tubers (4mg.) were pounded with whole plant of *Tinosporia cordifolia* (4mg.), boiled and decotation was prepared. This decoction (2

				ml.) was given with honey twice a day for a period of five to six days.
	Curcuma longa L.	R	Oral	The rhizome pounded and mixed with ghee and milk. This was given to the patient to drink for two days.
	Cuscuta reflexa Roxb.	WP	Topical	The whole plant was burnt on fire and fumes were inhaled to cure body pain in fever.
	Calotropis procera (Ait) R. Br.	La	Oral	The latex (1ml.) was mixed with sugar (10 ml.) and pellets were made. These pellets were given orally with warm water for two times. Excess of milk given to the patients to make drug more effective.
	Ocimum sanctum L.	LE	Oral	The leaves were crushed and boiled with water and this infusion (1ml) was given with warmed water within duration of 4 hours.
	Tinosporia cordifolia (Willd.) Miers. ex. Hook. F. & Thoms	WP	Oral	The whole plant (17 gm.) of *T. cordifolia*, leaves of *S. nigrum* and root of *Cyperus rotundus* (4gm.) dipped in water for whole night and was boiled in the morning and filtered. This filterate was given for two times a day.
Fistula	*Calotropis procera* (Ait) R. Br.	La	Topical	The latex of the plant was mixed with root powder of *Curcuma longa* and this paste was applied on the fistula for five days.
	Cocculus villosus DC.	R	Topical	The root of the plant rubs on the stone. After rubbing the paste applied topically on the fistula to accumulate the pus.
	Ficus benghalensis L.	LE	Topical	The leavers were pounded and mixed with equal amount (8 gm. each) of root of *Borrahavia dffusa* and leaves of *Tinospora cordafolia* and the paste was applied on fistula for three days.
	Opuntia dillenii (Ker- Gawl.) Haw.	LE	Topical	The warmed dethroned leaf was wrapped topically around the affected part for one month.
	Peperomia pellucida (L.) Kunth	LE	Topical	Slightly warm leaves were used for blistering surface.
Eye diseases	*Albizia lebbeck* (L.) Benth.	LE	Local (eye)	The leaves were mixed and pounded with *Tamarindus indica* (leaves), *Curcuma longa* (rhizome), *Punica granatum* (leaves). A band of these herbs was applied on the eye to cure eyes etching.
	Argemone mexicana L.	LE	Local (eye)	The leaves were pounded and mixed the cow ghee. The band of this poultice was wrapped around the eyes to cure pain in the eye.
	Calotropis procera (Ait) R. Br.	La	Local (eye)	The surma was prepared from the latex, by dipping the cotton in the latex for seven times and sun dried. This cotton was burnt on fire and this surma was applied in the eye.
	Cassia obtusifolia L.	F	Local (eye)	Dry powder of fruit (2 mg.) was used for the preparation of black surma which was used to cure itching of eye.
	Curcuma longa L.	R	Local (eye)	The rhizome was pounded and mixed with alum, leaves of *Tamarindus indica*, and leaves of *Albizzia lebbeck* (6 gm. each). The poultice of all parts was made and wrapped around the eye to cure pain in eye.

	Emblica officinalis Gaertn.	F	Local (eye)	The fruit juice (4 drops) was used for eye-washing of swelled eye.
Jaundice	*Acacia arabica* (Lam.) Willd.	FL	Oral	The flower (8gm.) pounded and mixed with 2 gm. of sugar. The drug was given thrice a day.
	Emblica officinalis Gaertn.	F	Oral	The fruit juice was mixed with powder of *Curcuma longa* (rhizome) and 2 ml was given twice a day.
	Ficus benghalensis L.	LE	Oral	The paste of 3 - 4 fresh leaves was prepared by mixing with sugar and water. This was given twice a day for 7 days.
	Punica granatum L.	F	Oral	The dry fruit grains (10 gm.) were dipped in 500ml. of water for 30-35 minutes. Grains were rubbed in water and the mixture was filtered and adds the sugar in it. This was given in morning and evening time for a week.
	Tinospora cordifolia (Willd.) Miers. ex. Hook. F. & Thoms	WP	Oral	The whole plant (10- 20 gm.) pounded and mixed with sugar. 1 teaspoon of the powder given orally to eliminate toxins of liver.
Male fertility disorders	*Acacia arabica* (Lam.) Willd.	FR, G	Oral	The pods (10 gm.) were mixed with gum of *Acacia arabica*, rhizome of *Asparagus racemosus* and isabgol and were pounded. This powder (10 mg.) was given twice a day to concentrate the semen.
	Albizia lebbeck (L.) Benth.	SE	Oral	The seeds (6 gm.) mixed with seeds of *Tamarindus indica* (6 gm.) and pounded together and paste was made after mixing with latex of *Ficus benghalensis* to prepare the pellets. These pellets were given thrice a day to cure male infertility.
	Calotropis procera (Ait) R. Br.	LE	Oral	The fresh leaves (100 gm.) were boiled in water for one hour and mixed 1 kg. cow ghee. Give this decoction with milk to increase seminal strength.
	Datura metel L.	SE	Oral	The seeds mixed with *Anacyclus pyrethrum* and *Syzygium aromaticum* and the pellets were prepared with seed oil of *Azadirachta indica. Given twice a day* to increase sex urge in male.
	Ficus benghalensis L.	LE	Oral	The fresh leaves (10 gm.) were shade dried and pounded. This powder was administrated orally with cow milk in morning and evening time to increase male strength and for conception.
	Momordica dioica Roxb. (Ex willd.)	R	Oral	The root of male plant was pounded and infusion was prepared after boiling with water. This infusion was given orally for one month to increase male fertility.
	Ocimum sanctum L.	WP	Oral	Male potency can be increased with oral intake of 1-2 gm of powder of plant with jaggery taken for 4 weeks.
	Pedalium murex L.	F	Oral	The fruit of plant boiled with water and this infusion (2ml) was given with cow ghee to increase masculine strength, cure impotence and also prevent nocturnal emission.
Mental	*Argemone*	R	Local	The juice of root mixed with equal part of seeds of Cannabis sativa and paste was made with cow urine. One or two drop of this paste poured in

Disorders	*mexicana* L.		(nose)	nose to cure epilepsy.
	Cannabis sativa L.	LE, WP	Oral	The leaves and whole plants (200mg) were pounded and the powder was taken with *Ferula assafoetida* (hing) (2mg.) for one month to cure the hyperactivity.
	Datura metel L.	SE	Oral	The seeds were pounded and boiled with water to make infusion and this infusion (2 ml) was given to cure the psychiatric patients.
	Mesua ferrea L.	SE	Local (nose)	The seeds (4gm.) mixed with whole plant of *Cuscuta reflexa* (6 gm.) and seeds of *Datura metel*((4gm.). The mixture was boiled in water in the morning. Add juice of *Cuscuta reflexa* (2ml.) and *oil* of *Brassica juncea* (1 ml.) in it. 2 or 3 drops were poured in the nose to cure epilepsy
Microbial contaminations	*Allium sativum* L.	C	Oral	Chewed 1 raw peeled cloves of garlic in a day to kill mouth bacteria.
	Cyperus rotundus L.	T	Oral	The tuber of plant pounded with fruit of *E. officinalis*, fruit of *T. bellirica* and fruit of *T. chebula*, 10 gm. each and these were boiled in 500ml of water for three hours. The decoction given to the patients orally twice a day.
	Melia azadirachta L.	SB	Oral	Powdered stem bark (50 gm.) was boiled in 500ml of water for three hours and decoction (3ml) given to the patients for twice a day to kill intestinal bacteria
Mouth ulcers	*Acacia catechu* (L. f) willd.	SB	Local (mouth)	The stem bark (2mg.) was pounded and paste was made with castor oil (1ml.) and applied on the ulcers of mouth for 3 days.
	Cordia dichotoma Forst. f.	F	Local (mouth)	The mucilaginous part of fruit was applied on mouth ulcers for three days
Piles	*Aloe vera* (L.) Burm.f.	LE	Topical	The poultice of warm peel of leaf wrapped on the pile for three days.
	Capparis aphylla Roth.	LE	Topical	The leaves were pounded and paste was prepared after mixing with cow ghee and oil of *Seasamum indicum*. This paste was applied to the bleeded piles for 5 days.
	Melia azadirachta L.	FL	Oral	The flowers pounded with seeds of *Azadirachta indica*, and flower juice of *Tagetes erecta.* Pellets were made and theses pellets were given in the morning for ten days.
	Raphanus sativus L.	SE	Topical	The seeds (1 gm.) were pounded with seeds of *Brassica juncea* (1 gm.), seeds of *Crotalaria juncea* and than filtered. The filtrate was mixed with cow ghee and this poultice was banded to the affected part for 4 or 5 days.
Respiratory problems	*Datura metel* L.	LE	Local (nose)	The leaves (5 gm.) were mixed with leaves (5 gm.) of *Barleria prionitis* and fumigated together. The inhalation of these fumes can cure respiratory problems

	Oroxylum indicum (L.) Vent.	SB	Oral	1 gm. of the stem bark powder was mixed in the ginger powder and was taken with honey for twice a day.
Skin diseases	*Argemone mexicana* L.	SE	Topical	Mixed the seeds oil (2.5 lt.) with carbonic acid (1gm.). This paste was applied topically after adding *Ricinus communis* oil (4 times) in it to cure skin irritation.
	Azadirachta indica A. Juss.	SE	Oral	Seeds (60gm.) were pounded with 10 gm. of sugar and pellets were prepared. Oral administrations of these pellets were given thrice a day to cure skin boils.
	Cassia fistula L.	LE	Topical	Made a paste of 10- 15 fresh leaves and apply two times for four days to cure itching of skin.
	Calotropis procera (Ait) R. Br.	La	Topical	The latex (4ml.) mixed with black cumin (4gm.), seed oil of *Brassica juncea* and water (2.5 lt.). Boiled this mixture for 2 hours to make a decoction. Filter this decoction and apply topically on the affected part to cure skin diseases for one month.
	Ocimum basilicum L.	LE	Topical	The paste of leaves of mixed with lemon juice and applied topically to cure the face acne.
	Mangifera indica L.	F	Topical	The unripe fruit juice was extracted and adds with methylated spirit. Applied topically on the skin to cure skin mark.
Snake bite	*Achyranthes aspera* L.	LE	Topical, Oral	The fresh leaves (8- 10) were pounded and paste was prepared. The poultice of this paste wrapped topically on the snake bite area to prevent microbial contamination. An infusion made by boiling with water also administrated orally (5 ml) to initiate vomiting to expeled snake venom
	Allium cepa L.	R	Topical, Oral	The root juice (10 ml) was administrated orally to the patients instead of water (in case of non poisonous snake bite). Extracted juice was also applied topically to the affected part to prevent irritation in the wound.
	Albizia lebbeck (L.) Benth.	RB,FL ,SE, SB	Oral	The seeds, root bark, flower and stem bark (1gm. each) were pounded together and make infusion with water and given orally (5ml.) three times a day.
	Artemisia scoparia Waldst. & Kit.	WP	Oral	The whole plant was pounded and boiled with water. This infusion (2 ml) was administrated orally to the victim for three to five times to neutralize the snake venom.
	Azadirachta indica A. Juss.	LE	Oral	The leaves (50 gm.) pounded and boiled with water for 30 minutes. Oral administration of this infusion (5 ml) was given to identify the type of snake venom (poisonous or non poisonous). If its taste appear sweet to the victim than snake was poisonous. The oral infusion (5ml) with cow ghee was also given if victim demands water.
	Butea monosperma (Lam.) Taub.	RB	Oral	The root bark was pounded and boiled with water to make infusion. This infusion was given (oral tract) according to body weigh ranging from 4 to 10 ml. This infusion will neutralize the snake venom without initiating vomiting and diarrhea in the victim.
	Cassia fistula L.	FR	Oral	The fruit (2gm.) pounded and mixed with seeds oil of *Azadirachta indica* (2 gm.), black pepper (2 gm.).The pellets were prepared from this mixture and given to victim within three hours duration. Cow ghee and

			black pepper were also given to the victim after these pellets. This drug was very effective against poisonous snake.
Calotropis procera (Ait) R. Br.	LE	Topical, Oral	The leaves of *Cannabis sativa*, white alum and potash were pounded together and mixed with the shade dried latex of *Calotropis procera*. The poultice of this paste was applied topically to the snake bite site after making a cut at the site. And if the snake venom spread in the victim body than 1gm. of the paste was given by dissolving in water to elute the venom through vomiting. Put 2-3 drops of this infusion in nose of victim to break the unconsciousness. The drops of latex can also be applied topically at snake bite site to neutralize the snake venom.
Cannabis sativa L.	LE	Oral	The leave were pounded and boiled with water to make infusion and give this infusion (6ml.) to victim for three days. The paste poultice can also be applied on the snakebite site to neutralize the venom.
Cassia occidentalis L.	R	Oral	The root pounded and mixed with black pepper, cow ghee and the pellets were prepared. These pellets (2 per day) were given to the victim to neutralize the snake venom.
Citrullus colocynthis (L.) Schrad.	R	Oral	The infusion made after boiling the root (5 gm.) with water given to the victim for identification of the snake. Snake seems to be poisonous if infusion tastes sweet.
Cocculus villosus DC.	R	Oral	The infusion of root made after boiling it with water and administrated (5 ml) thrice a day for two days to the victim.
Eclipta alba (L.) Hassk.	LE	Topical, Oral	The leaves were pounded and boiled with water to make infusion. This infusion (4ml.) given to the victim in case of blood oozes out of nose and mouth. The victim should not be allowed to sleep. The paste poultices can also be wrapped on the snake bite site to accumulate the poison at the centre.
Ficus benghalensis L.	R	Oral	The shade dried roots were pounded and filtered through muslin cloth and store this powder in a bottle. Oral administration of 6gm. mixed with cooper sulphate can be given to the victim within duration of 1 hour to initiate the vomiting. The snake venom will come out through vomiting.
Gloriosa superba L	F, R,	Oral	The fruit and root (10 gm. each) were pounded and mixed with leaves of *Achyranthes aspera* (8gm.), leaves of *Azadirachta indica* (5 gm.), leaves of *Cannabis sativa* (4 gm.), fruit of *Citrullus colocynthis* (5 gm.), fruit of *Momordica balsamina* (4 gm.) and latex of *Calotropis procera* (8 ml.). Boiled all these drugs in water (5 lt.) for one hour to make decoction. Filtered it and cooled filterate (5ml.) was given intermetantly within duration of three hours to the victim to expeled the poision through vomiting and diaorrhea. This decoction is best remedy in case of posionous snake bite.
Kyllinga monocephala Rottb	R	Oral	The roots (10gm.) were pounded and mixed with cow ghee (2gm.) and given to victim to remove the remaining part of poison.
Leucas cephalotes Spreng	WP	Oral	The juice of the whole plant was extracted. Put (5- 6 drops) in both eyes to prevent blindness. 5 ml. of the juice was given to drink to neutralize the venom. Precaution must be taken that never give water with *Leucas*

				cephalotes.
	Momordica balsamina L	F	Oral	The fruits were pounded and boiled with water to make the infusion. This infusion (5ml.) was given to victim to neutralize the snake venom.
	Ocimum sanctum L.	LE,R	Topical, Oral	In case of non poisonous snake bite give 3 ml of leaves juice within the duration of three hours and the poultice of root paste can be wrapped at the bite site to reduce the pain.
Tooth ache	*Achyranthes aspera* L.	LE	Local (mouth)	The leaves (2-3) were pounded and juice extracted. This juice was applied topically on the tooth with cotton plug.
	Argemone mexicana L.	WP	Local (mouth)	The whole plant was boiled with water and the infusion was filtered. Gargle with this infusion for three times a day to relieve tooth ache.
	Cassia fistula L.	F	Oral	The decoction of fruit prepared after boiling the fruit for 1 hour and applied topically on tooth.
	Ficus benghalensis L.	La	Local (mouth)	The latex of the plant soaked in cotton and applied topically on the tooth.
Wound healing	*Argemone mexicana* L.	La	Topical	Applied topically the latex of the plant on the wound for healing.
	Bryophyllum calycinum Salisb.	LE	Topical	The poultice of leaves paste can be wrapped topically (for two days) around the wound to subside swelling.
	Cuscuta reflexa Roxb.	WP	Topical	The juice of fresh plant applied on the wound for one week for sterilization of wound.
	Calotropis procera (Ait) R. Br.	RB	Topical	The root bark pounded and mixed with powder of *Triticum aestivum* and jaggery and to make a paste. The poultice of this paste was applied topically to the wound to cure pain in the wound.
	Ficus benghalensis L.	SB	Topical	A paste of powdered stem bark was made after mixing with cow ghee, oil of *Sesamum indicum* and applies topically to stop bleeding in wound.

Plant parts used: C=Cloves, G=Gum, FL=Flower, F=Fruit, SE=Seeds, LE=Leaves, R=Rhizome, Root, WP=Whole plant, La=Latex, SB=Stem bark and RB=Root bark
